# Di(2‐Ethylhexyl) Phthalate Exposure and Risk of Diabetic Kidney Disease: Epidemiological Association and Mechanistic Insights

**DOI:** 10.1002/dmrr.70209

**Published:** 2026-07-28

**Authors:** Dapeng Yin, Yan Li, Wei Wu, Xiaojuan Wang, Junhua He, Yikun Zhu, Jin Li

**Affiliations:** ^1^ Division of Endocrinology Second Hospital of Shanxi Medical University Taiyuan Shanxi China; ^2^ Division of Respiratory Second Hospital of Shanxi Medical University Taiyuan Shanxi China

**Keywords:** DEHP, diabetic kidney disease, lipid metabolism, molecular docking, network toxicology

## Abstract

**Objective:**

This study aims to explore the association between exposure to di‐(2‐ethylhexyl) phthalate (DEHP) and diabetic kidney disease (DKD), and to elucidate its potential molecular mechanisms. The study findings provide scientific evidence for the prevention and control of environmental risk factors related to DKD.

**Methods:**

We utilised the United States National Health and Nutrition Examination Survey (NHANES) database to evaluate the correlations of DEHP exposure with DKD risk and renal function indices. Candidate targets were screened using network toxicology, and functional enrichment analysis was conducted. The binding ability of DEHP to the candidate mechanistic targets was verified through molecular docking. The toxicity and target effects of DEHP and its metabolites were verified using HK‐2 cells.

**Results:**

The NHANES analysis demonstrated a positive correlation between DEHP exposure and the risk of DKD, with a significant dose‐response relationship observed. Network toxicology identified ALB, ESR1, and MMP9 as candidate mechanistic targets in DEHP‐induced DKD. In vitro experiments confirmed that DEHP and its metabolite MEHP reduced HK‐2 cell viability, induced lipid deposition, and altered the expression of candidate mechanistic targets.

**Conclusion:**

DEHP is an important environmental risk factor for DKD. It may promote the development and progression of DKD by regulating key targets and interfering with lipid metabolism.

## 1 Introduction

Di(2‐ethylhexyl) phthalate (DEHP) is among the most prevalent plasticisers, extensively utilised in polyvinyl chloride products, food packaging, medical devices, personal care items, and building materials. Due to the non‐covalent bonding between DEHP and the plastic matrix, it is highly unstable and prone to migration and release into the environment [1]. The released DEHP enters the human body through skin contact, the digestive tract and the respiratory tract, and is rapidly metabolised into more toxic metabolites, such as mono(2‐ethylhexyl) phthalate (MEHP) [2, 3]. The elimination half‐life of these metabolites in the human body can range from 15 to 24 h, and long‐term low‐dose exposure can lead to continuous accumulation [4]. Consequently, DEHP has been frequently detected in human blood, urine, breast milk, and amniotic fluid [5, 6, 7]. As a prevalent endocrine disruptor, DEHP has been associated with chronic diseases, including diabetes, liver diseases, and cardiovascular diseases, according to multiple studies [8]. However, there is a scarcity of research on its impact on diabetic microvascular complications, particularly diabetic kidney disease (DKD). The existing studies only provided preliminary epidemiological clues regarding the association between DEHP exposure and renal function impairment [9, 10]. Systematic research on the association between DEHP and DKD is imperative.

DKD is one of the serious microvascular complications of diabetes mellitus and a major cause of end‐stage renal disease. Epidemiological studies indicate that approximately 40% of individuals with diabetes worldwide will develop DKD [11]. The presence of DKD substantially elevates the risk of cardiovascular events and all‐cause mortality among diabetic patients, thereby imposing a considerable societal burden. The pathogenesis of DKD is complex. It is caused by damage to the glomerular filtration barrier, podocyte apoptosis and tubulointerstitial fibrosis due to long‐term hyperglycemia. It is jointly regulated by environmental factors, epigenetic modifications and inflammatory pathways [12, 13]. Recent research has increasingly focused on the contribution of environmental pollutants to DKD pathogenesis. Studies indicate that concentrations of DEHP and its metabolites are significantly elevated in diabetic patients compared to healthy individuals, with exposure levels showing a significant positive correlation with blood glucose levels and markers of renal function impairment [14, 15]. With the increasing prevalence of diabetes and the widespread use of plastic products, long‐term low‐dose exposure to DEHP has become a significant public health issue that cannot be ignored. However, the underlying molecular toxicological mechanism remains unclear.

Therefore, this study first utilised data from the National Health and Nutrition Examination Survey (NHANES) to investigate the epidemiological association between DEHP and DKD. A ‘DEHP‐targets‐disease’ regulatory network was then constructed using network toxicology methods to identify potential targets and signalling pathways. Molecular docking was further used to simulate the binding modes of DEHP with key targets, and changes in the expression of candidate mechanistic targets were validated through in vitro experiments. The objective of this study was to elucidate the potential pathogenic mechanisms of DEHP in DKD and to provide a scientific basis for evaluating the renal toxicity risk posed by DEHP.

## 2 Materials and Methods

### 2.1 Research Population

This is a cross‐sectional study using data from the NHANES database (2003–2018) in the United States. NHANES uses a multi‐stage, stratified probability sampling design, and its data reflect the health and nutritional status of all residents in the United States. All participants signed written informed consent forms and received approval from the Ethics Review Committee of the National Centre for Health Statistics (NCHS) of the United States. A total of 80,312 participants from the NHANES database between 2003 and 2018 were initially included in the study. Firstly, 35,522 people under the age of 20 were excluded, resulting in 44,790 adults. Subsequently, 32,665 participants were excluded due to the lack of data on urinary DEHP metabolites (MEHP, MEHHP, MEOHP, MECPP) and urinary creatinine (Ucr). The remaining 12,125 participants were included. Finally, 9976 individuals with missing eGFR, urine albumin‐to‐creatinine ratio (ACR), pregnant women, type 1 diabetes, or missing survey weights were excluded. A total of 2149 participants were ultimately included in the analysis. Supporting Information S1: Figure S1 shows the screening process for the research subjects.

### 2.2 Exposure and Results

Urine samples were analysed at the National Environmental Health Centre. The urinary metabolites of DEHP, including MEHP, mono(2‐ethyl‐5‐hydroxyhexyl) phthalate (MEHHP), mono(2‐ethyl‐5‐oxohexyl) phthalate (MEOHP), and mono(2‐ethyl‐5‐carboxypentyl) phthalate (MECPP), are recognised as sensitive indicators of DEHP exposure [16]. To minimise the impact of urine dilution, metabolite concentrations were adjusted for Ucr and reported as ng/mg crt. Based on previous studies, a ∑DEHP summary index was established to integrate exposure levels across multiple DEHP metabolites [17]. The calculation method was to convert the concentrations of each DEHP metabolite to molar concentrations, then multiply by the molecular weight of the DEHP parent molecule to obtain the total exposure level. Finally, a natural logarithmic transformation was applied to obtain approximately normal distributions. Based on the weighted distribution of ln∑DEHP, the study subjects were divided into three quartiles for subsequent statistical analysis.

The diagnosis of diabetes is based on the American Diabetes Association standards and is determined by combining questionnaire information [18]. Any of the following conditions can confirm the diagnosis: glycosylated haemoglobin (HbA1c) ≥ 6.5%, fasting blood glucose ≥ 7.0 mmol/L, 2 h blood glucose after oral glucose tolerance test ≥ 11.1 mmol/L, random blood glucose ≥ 11.1 mmol/L, self‐diagnosis of diabetes, or current use of insulin and other hypoglycemic drugs. Under the premise of confirmed diabetes, the diagnosis of DKD is based on a ACR ≥ 30 mg/g or an estimated glomerular filtration rate (eGFR) < 60 mL/min/1.73 m^2^ [19].

### 2.3 Covariates

Based on previous studies and clinical experience, we selected several important covariates, including gender, age, body mass index (BMI), race, marital status, education level, poverty income ratio (PIR), alcohol history, smoking history, hypertension, hyperlipidaemia, metabolic syndrome, and cardiovascular diseases. Laboratory measurement indicators include: white blood cells (WBC), haemoglobin (Hb), platelets (PLT), alanine aminotransferase (ALT), aspartate aminotransferase (AST), total cholesterol (TC), triglycerides (TG), HbA1c, and Ucr. Missing values were filled in using multiple imputation methods. Based on the mice package in R software, the chained equations method was applied with five imputations to impute missing values and control for bias caused by missing data.

### 2.4 Collection of DEHP and DKD Targets

The SMILE format of DEHP was obtained from the PubChem database (https://pubchem.ncbi.nlm.nih.gov/). Subsequently, potential DEHP targets were retrieved through the SwissTargetPrediction database (https://swisstargetprediction.ch/), the Similarity ensemble approach database (https://sea.bkslab.org/), and the PharmMapper database (https://www.lilab‐ecust.cn/pharmmapper/index.html) [20]. We combined the obtained targets to construct a DEHP target dataset. Use ‘diabetic nephropathy’ and ‘diabetic kidney disease’ as keywords to identify DKD‐related targets. Retrieve relevant targets from the Genecards database (https://www.genecards.org/, relevance score ≥ 10) and the Comparative Toxicogenomics Database (https://ctdbase.org/, inference score ≥ 10) [21]. Then, we combined these targets into a DKD target dataset. Taking the intersection of the DEHP and DKD targets to obtain the common targets of DEHP and DKD.

### 2.5 Construction of Protein‐Protein Interaction (PPI) Network

After the common genes between DEHP and DKD were identified, a PPI network was constructed using the STRING database (https://cn.string‐db.org/). To ensure high network confidence, an interaction score threshold of > 0.4 was applied. Visualisation and network topology analysis were subsequently performed using Cytoscape software (v 3.10.0). Four algorithms, including Maximal Clique Centrality (MCC), Maximum Neighbourhood Component (MNC), Closeness Centrality, and Bottleneck Centrality, were utilised to identify the core proteins in the network [22]. The intersection of the core proteins identified by these four algorithms was then used to define the key target of DEHP involved in DKD.

### 2.6 Gene Ontology (GO) and Kyoto Encyclopaedia of Genes and Genomes (KEGG) Enrichment Analysis

To elucidate the mechanism by which DEHP induces DKD, GO and KEGG enrichment analyses were conducted. Functional enrichment analysis of the common genes between DEHP and DKD was performed using the DAVID database (https://davidbioinformatics.nih.gov/). The GO analysis included three categories: biological process (BP), cellular component (CC), and molecular function (MF). The top 15 results were selected after false discovery rate (FDR) correction with adjusted *p* < 0.05.

### 2.7 Molecular Docking of DEHP With Candidate Mechanistic Targets

In this study, molecular docking was used to predict the binding affinity between DEHP and the candidate mechanistic targets. DEHP was used as the ligand and its structure was obtained from the PubChem database (PubChem CID: 8343). The three‐dimensional crystal structures of oestrogen receptor 1 (ESR1, PDB ID: 1SJ0), matrix metallopeptidase 9 (MMP9, PDB ID: 1GKC), and albumin (ALB, PDB ID: 1AO6) were obtained from the RCSB PDB database (https://www.rcsb.org/). Before docking, the receptor was pretreated using PyMol software, including removing water molecules and all heteroatoms (including small molecules, ions, and cofactors), and adding polar hydrogen atoms. The preprocessed receptor structure was saved as a PDB file. Subsequently, blind docking was performed using the CB‐Dock2 platform (http://183.56.231.194:8001/cb‐dock2/index.php) [23]. The CB‐Dock2 algorithm, based on curvature, automatically identifies potential binding pockets on the protein surface and invokes AutoDock Vina to perform molecular docking calculations. Meanwhile, charge assignment and three‐dimensional conformation generation were performed for the ligand. For each receptor–ligand complex, multiple docking conformations were generated, and the conformation with the lowest Vina score (binding free energy, kcal/mol) was selected as the optimal binding mode, with the corresponding binding energy recorded [24]. Finally, the docking results were visualised using the PyMol software.

### 2.8 Exploring the Association Between DEHP and DKD Through In Vitro Experiments

The human renal tubular epithelial cell line HK‐2 (Procell, Wuhan, China) was cultured in MEM containing 10% foetal bovine serum and 1% penicillin/streptomycin. DEHP (CAS No. 117‐81‐7) and MEHP (CAS No. 4376‐20‐9) were purchased from Macklin Biochemical Co. Ltd. (Shanghai, China) and dissolved in dimethyl sulfoxide (DMSO). HK‐2 cells were treated with 30 mM glucose combined with 300 μM palmitic acid (HG/PA) for 24 h to establish an in vitro model of DKD [25, 26]. A normal glucose control (NG Control, 5.6 mM glucose) group and an isotonic control group (5.6 mM glucose + 24.4 mM mannitol) were set up to exclude the effect of osmotic pressure. Then, Oil Red O staining was used to observe the lipid deposition of cells to verify the validity of the model. The effects of DEHP and MEHP on the viability of HK‐2 cells were evaluated by cell counting kit‐8 (CCK‐8) assay (NCM Biotech, Suzhou, China). Based on the cell viability and previous research reports, 100 and 200 μM DEHP were selected for intervention [27]. Simultaneously, parallel experiments were conducted using low and high doses of MEHP that matched the toxicity level of DEHP. Meanwhile, 0.1% (v/v) DMSO was added to each group to eliminate solvent interference. After 24 h of incubation, we performed Oil Red O staining to assess intracellular lipid accumulation and quantify the positive staining area. The mRNA expressions of ALB, ESR1 and MMP9 were also detected.

### 2.9 Reverse Transcription Quantitative Real‐Time PCR (RT‐qPCR)

Total RNA was extracted from cells using TRIzol reagent (Invitrogen, USA). RNA concentration and purity were assessed using a NanoDrop spectrophotometer (Thermo Fisher Scientific, USA). Complementary DNA (cDNA) was synthesised via reverse transcription using PrimeScript RT (Takara, Japan). According to the instructions, the qPCR reaction was performed using SYBR Premix Ex Taq II (Takara, Japan) on a LineGene 9600 Plus (Bioer Technology, Hangzhou, China). The expression level of the housekeeping gene β‐actin was used as the internal reference control, and the relative expression level of the target gene was calculated using the 2^−ΔΔCt^ method. Primer sequences are provided in Supporting Information S1: Table S1.

### 2.10 Statistical Analysis

All statistical analyses considered the complex multistage sampling design of NHANES. Sampling weights from NHANES were included in all analyses to correct for sampling bias and ensure nationally representative results.

Participants were divided into two groups based on whether they had DKD. Continuous variables were expressed as mean ± standard error, and the Student's *t*‐test was used for inter‐group comparison. Categorical variables were presented as frequencies (*n*) and percentages (%), and group differences were examined using the weighted chi‐square test. Furthermore, weighted multivariable logistic regression models were applied to evaluate the associations of urinary DEHP and its major metabolites (MEHP, MEHHP, MEOHP, MECPP) with DKD risk. Each metabolite was natural logarithmic transformation and categorised into tertiles. With the lowest exposure group (T1) as the reference, three stepwise correction models were constructed. Model 1 did not adjust for covariates; Model 2 adjusted for age, gender and race; and Model 3 further adjusted for BMI, marital status, education level, PIR, alcohol history, smoking history, hypertension, hyperlipidaemia, metabolic syndrome, and laboratory test indicators. The results were presented as weighted odds ratios (OR) along with corresponding confidence intervals (CI), and a trend test was conducted. Subsequently, a restricted cubic spline (RCS) was employed to explore the nonlinear association between DEHP and its main metabolites (MEHP, MEHHP, MEOHP, MECPP) and DKD. In addition, we conducted subgroup analyses and examined the effects of age, gender, race, hypertension, hyperlipidaemia, MetS and CVD. All analyses were performed using R software (version 4.4.2), and a *p* value < 0.05 was considered statistically significant.

For in vitro experiments, comparisons among multiple groups were performed using one‐way ANOVA followed by the LSD post hoc test. Oil Red O staining was analysed semi‐quantitatively using ImageJ software. Statistical analyses were performed using GraphPad Prism (version 9.5) software. A *p* value < 0.05 was considered statistically significant.

## 3 Results

### 3.1 Baseline Characteristics of Participants

A total of 2149 subjects were finally included in the analysis. Their baseline characteristics are shown in Supporting Information S1: Table S2. There were significant differences between the DKD and non‐DKD groups in age, marital status, education level, PIR, history of alcohol consumption, hypertension, MetS, CVD, Hb, PLT, ALT, TC, TG, HbA1c, and Ucr levels. Regarding environmental exposure, the ln∑DEHP levels of DKD patients were overall higher than those of non‐DKD patients. Although this difference did not reach statistical significance, the DKD population still showed a trend of higher DEHP exposure levels.

### 3.2 Correlation Between DEHP Metabolites and DKD

Three weighted logistic regression models were constructed to examine the associations of DEHP and its main metabolites (MEHP, MEHHP, MEOHP, MECPP) with DKD (Table 1). For DEHP, compared with the lowest exposure group (T1), the highest exposure group (T3) demonstrated a significantly increased risk of DKD (OR = 1.41, 95% CI: 1.02–1.94, *p* = 0.04), with a significant trend observed (*p* for trend = 0.035). In Model 2, which adjusted for age, gender, and race, the significance of the trend test weakened (*p* for trend = 0.068), but still showed a positive association. Model 3, which further adjusted for multiple potential confounding factors, indicated that the highest DEHP exposure remained significantly associated with DKD risk (OR = 1.48, 95% CI: 1.01–2.16, *p* = 0.04), and the trend test was significant (*p* for trend = 0.044). Further weighted logistic regression analysis of DEHP metabolites revealed that only MEHHP was significantly positively correlated with the risk of DKD. With increasing MEHHP concentrations, the risk of DKD increases. In model 3, the highest exposure group (T3) showed a significantly increased risk of DKD (OR = 1.50, 95% CI: 1.02–2.21, *p* = 0.04), and the trend test was also notable (*p* for trend = 0.041). However, other metabolites (MEHP, MEOHP, MECPP) were not significantly associated with DKD. These findings indicated that higher levels of DEHP and MEHHP exposure were associated with the risk of DKD.

**TABLE 1:** Association of DEHP and its metabolites with DKD.

Exposure	Model 1		Model 2		Model 3	
	OR (95% CI)	*p‐*value	OR (95% CI)	*p*‐value	OR (95% CI)	*p*‐value
ln∑DEHP ng/mg crt						
T1 (lowest)	Reference		Reference		Reference	
T2 (middle)	1.26 (0.94, 1.69)	0.12	1.27 (0.93, 1.73)	0.13	1.18 (0.83, 1.68)	0.36
T3 (highest)	1.41 (1.02, 1.94)	0.04	1.38 (0.97, 1.96)	0.07	1.48 (1.01, 2.16)	0.04
*p* for trend	0.035		0.068		0.044	
lnMEHP ng/mg crt						
T1 (lowest)	Reference		Reference		Reference	
T2 (middle)	1.08 (0.79, 1.49)	0.62	1.09 (0.78, 1.51)	0.61	1.23 (0.85, 1.76)	0.26
T3 (highest)	1.07 (0.77, 1.48)	0.69	1.13 (0.81, 1.57)	0.48	1.17 (0.82, 1.67)	0.37
*p* for trend	0.681		0.477		0.355	
lnMEHHP ng/mg crt						
T1 (lowest)	Reference		Reference		Reference	
T2 (middle)	1.23 (0.87, 1.74)	0.24	1.25 (0.87, 1.79)	0.22	1.16 (0.79, 1.69)	0.45
T3 (highest)	1.38 (0.98, 1.93)	0.07	1.42 (0.99, 2.04)	0.05	1.50 (1.02, 2.21)	0.04
*p* for trend	0.066		0.054		0.041	
lnMEOHP ng/mg crt						
T1 (lowest)	Reference		Reference		Reference	
T2 (middle)	1.22 (0.87, 1.71)	0.24	1.18 (0.83, 1.68)	0.35	1.05 (0.71, 1.55)	0.79
T3 (highest)	1.27 (0.91, 1.77)	0.16	1.22 (0.85, 1.75)	0.28	1.23 (0.83, 1.83)	0.30
*p* for trend	0.159		0.278		0.306	
lnMECPP ng/mg crt						
T1 (lowest)	Reference		Reference		Reference	
T2 (middle)	1.16 (0.87, 1.57)	0.31	1.10 (0.81, 1.50)	0.53	0.93 (0.64, 1.34)	0.68
T3 (highest)	1.31 (0.96, 1.78)	0.09	1.21 (0.86, 1.73)	0.27	1.28 (0.88, 1.87)	0.20
*p* for trend	0.086		0.274		0.206	

*Note:* Weighted logistic regression was used to estimate ORs and 95% CIs. Model 1 was unadjusted; Model 2 was adjusted for age, sex, and race; Model 3 was additionally adjusted for education level, marital status, PIR, alcohol, smoking status, BMI, CVD, hypertension, hyperlipidaemia, MetS, WBC, Hb, PLT, TC, TG, ALT, AST, HbA1c. Trend *p* values were calculated with T1 as the reference group.

### 3.3 Dose–Response Relationship Between DEHP and Its Metabolites and DKD

**FIGURE 1:**
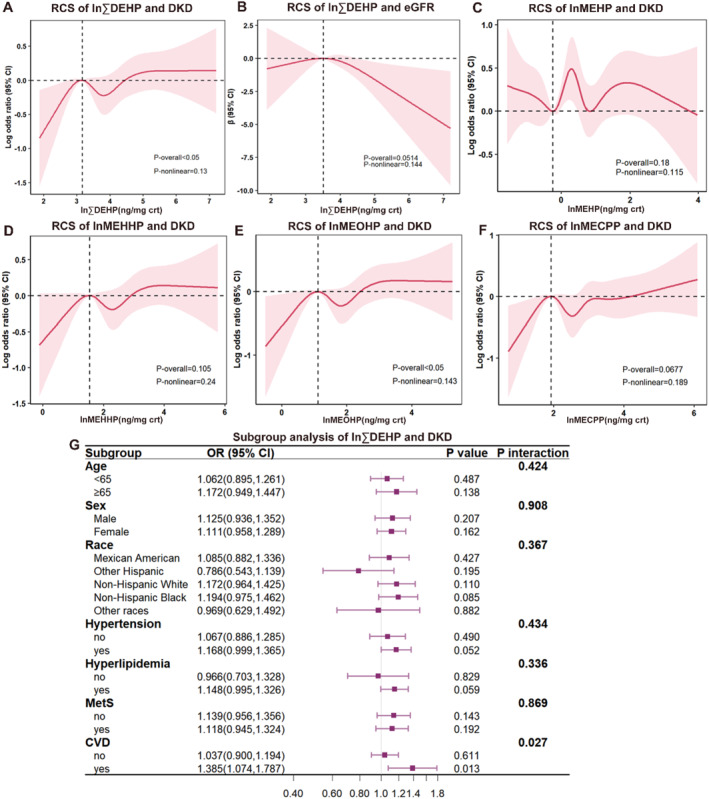
Dose–response and subgroup analyses of DEHP and its metabolites in relation to DKD risk. (A, B) RCS analyses of the associations between ln∑DEHP and (A) DKD risk and (B) eGFR. (C–F) RCS analyses of the associations between DKD risk and individual DEHP metabolites: (C) lnMEHP, (D) lnMEHHP, (E) lnMEOHP, and (F) lnMECPP. (G) Subgroup interaction analysis for the association between ln∑DEHP and DKD risk. DEHP, di‐(2‐ethylhexyl) phthalate; DKD, diabetic kidney disease; eGFR, estimated glomerular filtration rate; MECPP, mono(2‐ethyl‐5‐carboxypentyl) phthalate; MEHHP, mono(2‐ethyl‐5‐hydroxyhexyl) phthalate; MEHP, mono(2‐ethylhexyl) phthalate; MEOHP, mono(2‐ethyl‐5‐oxohexyl) phthalate; RCS, restricted cubic spline.

The RCS analysis revealed an overall positive correlation between ln∑DEHP and DKD (*p*‐overall < 0.05), with no significant nonlinear relationship observed (*p*‐nonlinear = 0.13) (Figure 1A). The spline curves showed a smooth trend between ln∑DEHP and DKD risk with relatively higher risk estimates at higher exposure levels. We further analysed the renal function indicators related to DKD. The results suggested that ln∑DEHP was approximately linearly negatively correlated with eGFR (*p*‐overall = 0.0514, *p*‐nonlinear = 0.144) (Figure 1B). Among all the metabolites, the overall association between lnMEOHP and the risk of DKD was statistically significant (*p*‐overall < 0.05), but there was no nonlinear relationship (*p*‐nonlinear = 0.143) (Figure 1E). No significant overall associations or non‐linear dose‐response relationships were observed for lnMEHP, lnMEHHP, and lnMECPP with the risk of DKD (Figure 1C,D,F). Overall, RCS analysis revealed similar dose‐response patterns between various DEHP metabolites and DKD.

### 3.4 Subgroup Analysis

Overall, no significant differences were observed in the association between ln∑DEHP and DKD across subgroups defined by age, gender, race, hypertension, hyperlipidaemia, and MetS. However, the association between ln∑DEHP and DKD changed with or without concurrent CVD status, with elevated ln∑DEHP levels associated with an increased risk of DKD (*p* = 0.027) (Figure 1G).

### 3.5 Acquisition of Target Genes

**FIGURE 2:**
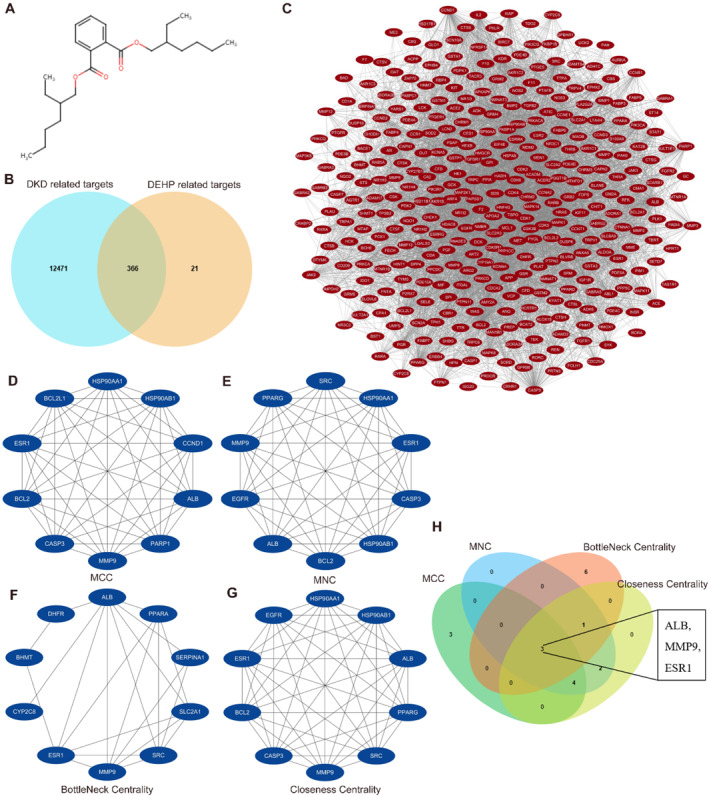
Identification of targets associated with DEHP and DKD. (A) Chemical structure of DEHP. (B) Venn diagram showing DKD‐related and DEHP‐related targets. (C) PPI network constructed from overlapping targets. (D–G) Identification of hub genes using the (D) MCC, (E) MNC, (F) BottleNeck centrality, and (G) closeness centrality algorithms implemented in Cytoscape. (H) Venn diagram showing the common hub genes identified by the four algorithms. DEHP, di‐(2‐ethylhexyl) phthalate; DKD, diabetic kidney disease; MCC, maximal clique centrality; MNC, maximum neighbourhood component; PPI, protein–protein interaction.

Figure 2A shows the secondary structure of DEHP. Subsequently, 387 DEHP target genes were identified from three databases (SwissTargetPrediction, Similarity ensemble approach, and PharmMapper). A total of 12,837 DKD‐related targets were retrieved from the GeneCards and Comparative Toxicogenomics Database. Finally, 366 common target genes were obtained (Figure 2B).

### 3.6 PPI Network of Common Genes and Candidate Targets

A PPI network was constructed for 366 common target genes using the STRING database (Figure 2C). Using the four algorithms (MCC, MNC, BottleNeck Centrality, and Closeness Centrality) in Cytoscape, the top 10 targets for each method (Figure 2D–G). The overlapping genes ALB, ESR1, and MMP9, identified by all four algorithms, were designated as candidate mechanistic targets for DEHP and DKD (Figure 2H).

### 3.7 GO and KEGG Pathway Enrichment Analysis

**FIGURE 3:**
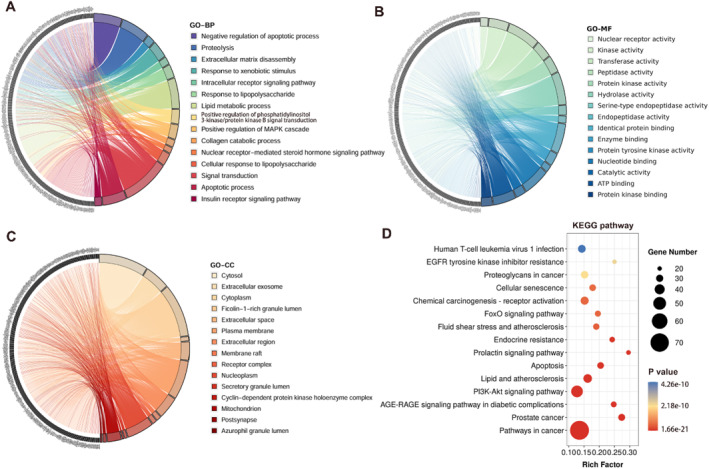
Functional enrichment analysis of the overlapping targets associated with DEHP and DKD. (A–C) GO enrichment analysis of the overlapping targets, showing the top enriched (A) biological process, (B) molecular function, and (C) cellular component terms. (D) KEGG pathway enrichment analysis of the overlapping targets. BP, biological process; CC, cellular component; DKD, diabetic kidney disease; DEHP, di‐(2‐ethylhexyl) phthalate; GO, gene ontology; KEGG, Kyoto encyclopedia of genes and genomes; MF, molecular function.

To elucidate the biological mechanisms underlying DEHP and DKD, we conducted a functional enrichment analysis of the shared genes. The GO enrichment analysis revealed that the common genes were mainly enriched in processes related to lipid metabolic process, positive regulation of phosphatidylinositol 3‐kinase‐protein kinase B (PI3K‐Akt) signal transduction, insulin receptor signalling pathway, xenobiotic stimulus response, and extracellular matrix disassembly during biological processes (Figure 3A). The molecular functions were mainly protein kinase and receptor binding activity, and the cellular components were mainly located in the cell membrane and extracellular regions (Figure 3B,C). The KEGG pathway enrichment indicated that the common genes were enriched in pathways such as lipid and atherosclerosis, the PI3K‐Akt signalling pathway, the AGE‐RAGE signalling pathway in diabetic complications, and chemical carcinogenesis—receptor activation (Figure 3D).

### 3.8 Molecular Docking of DEHP With Candidate Mechanistic Targets

**FIGURE 4:**
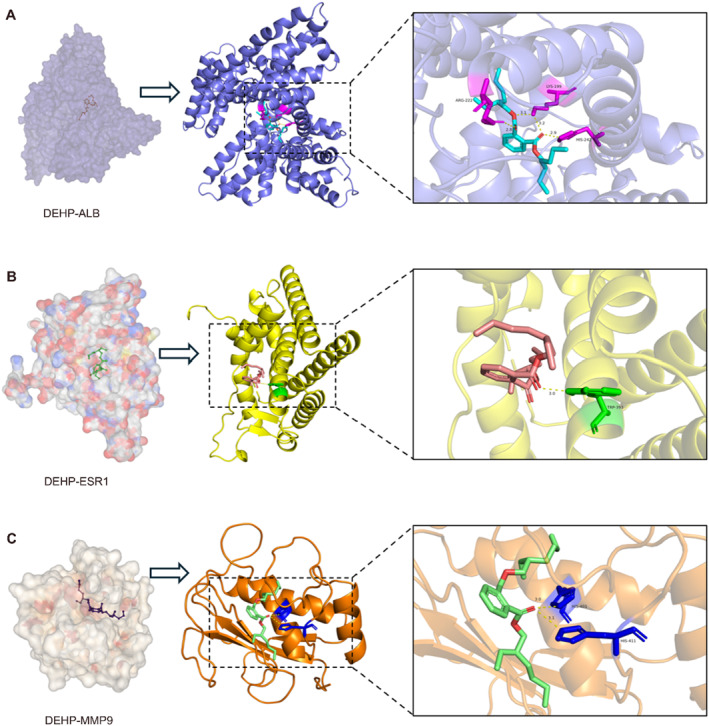
Molecular docking analysis of DEHP with candidate mechanistic targets. (A–C) Predicted binding modes of DEHP with (A) ALB, (B) ESR1, and (C) MMP9. The overall binding conformation and a magnified view of the binding site are shown. ALB, albumin; DEHP, di‐(2‐ethylhexyl) phthalate; ESR1, oestrogen receptor 1; MMP9, matrix metalloproteinase 9.

Molecular docking was used to study the interaction between DEHP and three candidate mechanistic targets (ALB, ESR1, and MMP9). Generally, a lower binding energy indicates a stronger interaction. The molecular docking results predicted that DEHP has a strong affinity for the three candidate mechanistic targets. The binding energies of DEHP with ALB, ESR1 and MMP9 were −6.9, −6.1 and −6.6 kcal/mol, respectively. These findings are presented in Figure 4A–C. The results suggested that these targets were integral to the molecular mechanism underlying DEHP‐induced DKD.

### 3.9 In Vitro Experiments Demonstrated the Role of the Candidate Mechanistic Targets

To determine whether the HK‐2 cell injury induced by HG/PA was due to metabolic toxicity rather than osmotic pressure changes, we set up different groups. As shown in Supporting Information S1: Figure S2, compared with the NG control group, there were no significant differences in lipid aggregation and the mRNA expressions of ALB, ESR1, and MMP9 in the isotonic control group (*p* > 0.05). The HG/PA group showed significant lipid deposition and upregulated MMP9 expression (Supporting Information S1: Figure S2). These results indicate that the in vitro DKD model was successfully established, and the observed cellular injury was primarily caused by metabolic stress rather than osmotic alterations.

**FIGURE 5:**
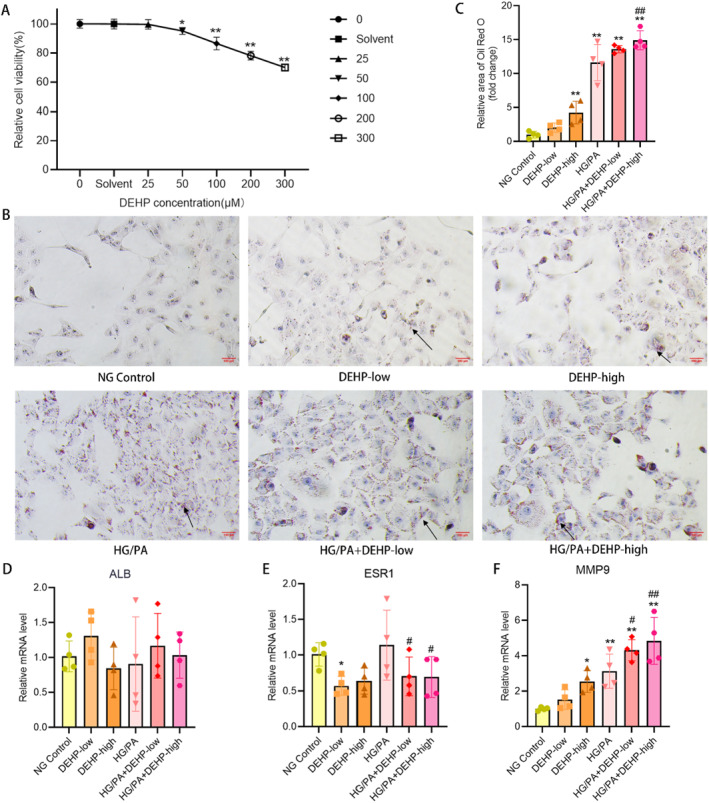
Effects of in vitro DEHP intervention on HK‐2 cells. (A) CCK‐8 assay of HK‐2 cell viability under DEHP intervention at different concentrations. (B) Oil red O staining showing the pro‐lipid accumulation effect of DEHP (scale bar = 100 μm). (C) Semi‐quantitative analysis of oil red O–positive staining area. (D–F) Relative mRNA expression levels of (D) ALB, (E) ESR1, and (F) MMP9 determined by RT‐qPCR following DEHP intervention. Data are presented as the mean ± SEM (*n* = 4). *p < 0.05, **p < 0.01 versus the NG control group; ^#^ *p* < 0.05, ^##^ *p* < 0.01 versus the HG/PA group. ALB, albumin; CCK‐8, cell counting kit 8; DEHP, di‐(2‐ethylhexyl) phthalate; DEHP‐high, DEHP 200 μM; DEHP‐low, DEHP 100 μM; ESR1, oestrogen receptor 1; HG/PA, 30 mM glucose + 300 μM palmitic acid; MMP9, matrix metallopeptidase 9; NG Control, 5.6 mM glucose.

To further verify the role of the candidate mechanistic targets in DEHP‐induced DKD, we conducted an in vitro experiment using HK‐2 cells. The CCK‐8 results indicated that the cell viability after treatment with 100 and 200 μM DEHP was 86.55% and 78.24%, respectively, and no significant cell death was induced (Figure 5A). Oil Red O staining showed that compared with the NG control group, DEHP could induce lipid aggregation in HK‐2 cells, and this effect was concentration‐dependent. Even in the in vitro DKD model induced by HG/PA, DEHP could further enhance the cell lipid aggregation (Figure 5B,C). The RT‐qPCR results indicated that, compared with the NG control group, DEHP decreased ESR1 expression and increased MMP9 expression, with no significant difference in ALB expression. Compared with the in vitro model of DKD induced by HG/PA, this difference in the changes of the candidate mechanistic targets induced by DEHP still existed (Figure 5D–F).

**FIGURE 6:**
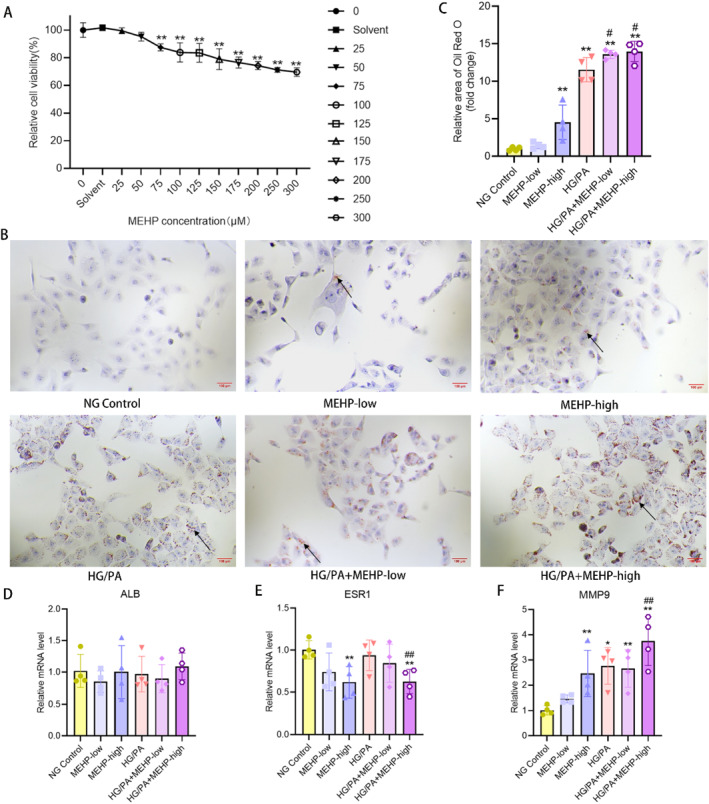
Effects of in vitro MEHP intervention on HK‐2 cells. (A) CCK‐8 assay of HK‐2 cell viability under MEHP intervention at different concentrations. (B) Oil red O staining of HK‐2 cells following treatment with MEHP alone or in combination with HG/PA (scale bar = 100 μm). (C) Semi‐quantitative analysis of lipid deposition area stained with Oil Red O. (D–F) Relative mRNA levels of (D) ALB, (E) ESR1, and (F) MMP9 detected by RT‐qPCR under MEHP intervention. Data are presented as the mean ± SEM (*n* = 4). *p < 0.05, **p < 0.01 versus the NG Control group; ^#^ *p* < 0.05, ^##^ *p* < 0.01 versus the HG/PA group. ALB, albumin; CCK‐8, cell counting kit 8; ESR1, oestrogen receptor 1; HG/PA, 30 mM glucose + 300 μM palmitic acid; MMP9, matrix metallopeptidase 9; MEHP, mono(2‐ethylhexyl) phthalate; MEHP‐high, MEHP 150 μM; MEHP‐low, MEHP 75 μM; NG Control, 5.6 mM glucose.

To verify whether the toxicity of DEHP mainly comes from its metabolite MEHP. We screened out the concentrations of MEHP that matched the toxicity of DEHP as 75 and 150 μM, respectively, by the CCK‐8 method (Figure 6A). Oil Red O staining showed that MEHP could also induce lipid deposition in HK‐2 cells, and the lipid deposition was more obvious at a higher concentration of MEHP (Figure 6B,C). Regarding the expression of candidate mechanistic targets, MEHP also increased the mRNA levels of MMP9 and decreased ESR1 expression, while ALB expression showed no significant change (Figure 6D–F). This suggests that MEHP could mimic the phenotypic effects of DEHP under equal toxicity conditions.

## 4 Discussion

DKD has emerged as the leading cause of end‐stage renal disease globally. The pathogenesis of DKD is complex, and to traditional factors such as high blood sugar and high blood pressure, increasing attention has been directed towards the role of environmental pollutants. DEHP is a widely present environmental pollutant that has been identified as a risk factor for various chronic diseases. This study systematically analysed epidemiological data from NHANES, combined with network toxicology analysis and in vitro experiments. This approach established a multidimensional evidence chain from population observation to molecular mechanisms. It deeply explored the relationship between DEHP and DKD as well as possible pathogenic mechanisms.

Our analysis based on the NHANES database revealed a significant positive association between DEHP exposure level and DKD risk. As DEHP exposure increased, the estimated eGFR demonstrated an approximately linear decline. The RCS analysis further confirmed that there was a clear dose‐response relationship between DEHP exposure and DKD, that is, higher DEHP exposure corresponded to greater DKD risk. These findings were consistent with previous studies, where DEHP exposure was closely associated with renal function impairment, and this impairment existed in both adults and children [28, 29]. Further analysis of DEHP metabolites revealed that the associations between different metabolites and the risk of DKD were not entirely consistent. Several factors may account for these discrepancies. Weighted logistic regression and RCS analysis differ in their statistical rationale and purposes. The former focuses on linear associations and risk effects, while the latter is designed to identify non‐linear dose‐response relationships. Such methodological differences can contribute to inconsistent findings for some metabolites. Meanwhile, DEHP is rapidly metabolised in the human body with a short half‐life, and its metabolic profile exhibits dynamic changes over time [30]. MEHP, as a primary metabolite, reflects recent exposure levels, while secondary metabolites such as MEHHP, MEOHP, and MECPP indicate long‐term cumulative exposure [31]. Varied sensitivities of statistical models to time‐specific exposure may lead to inconsistent results across metabolites. This also reflects the complexity of the distribution of environmental exposure levels in the population.

Network toxicology analysis provides clues to understand the underlying mechanism of DEHP involvement in the occurrence of DKD. We found that the overlapping targets of DEHP and DKD were mainly enriched in pathways closely related to diabetic complications, including lipid metabolism, the PI3K‐Akt signalling pathway, and the AGE‐RAGE signalling pathway. These pathways play an important role in the development of DKD. Specifically, lipid metabolism disorders are the core drivers of kidney damage in DKD. Excessive lipid deposition will cause lipid toxicity damage to the renal cells of the kidneys, aggravating inflammatory responses oxidative stress, etc. [32]. The in vitro experiments in this study confirmed that DEHP could cause significant lipid deposition in HK‐2 cells, thereby supporting the findings of the enrichment analysis. It was worth noting that the DEHP metabolite MEHP could also induce lipid deposition in HK‐2 cells. The selection is informed by the toxicological profile of MEHP, which serves as the primary hydrolysis product and principal active metabolite of DEHP. MEHP plays a pivotal role in the DEHP metabolic network and is a key mediator for the biological effects of DEHP [33]. Changes in its concentration can directly reflect the absorption and conversion efficiency of DEHP in the body and have good representativeness. Moreover, previous studies have demonstrated that MEHP induces epithelial‐mesenchymal transition in renal tubular epithelial cells, which promotes renal fibrosis and indicates a nephrotoxic effect [34]. Therefore, due to its critical metabolic role and well‐documented nephrotoxicity, MEHP was selected for further investigation and validation. In this study, MEHP showed similar toxicological effects to DEHP on cells, further supporting that DEHP exposure may jointly participate in the kidney injury process through its metabolites. This also strengthened the connection between epidemiological findings and mechanism research to a certain extent.

In this study, ALB, ESR1 and MMP9 were identified as the candidate mechanistic targets of DEHP. ALB is an important indicator of renal filtration function. In DKD, the increase in urinary albumin excretion rate directly reflects the structural or functional damage of the glomerular filtration barrier [35]. DEHP, as an endocrine disruptor, may broadly affect the transcriptional regulatory network of the kidneys and interfere with ESR1 expression [36]. This will weaken the antioxidant and anti‐inflammatory protective effects mediated by ESR1 and increase the susceptibility of kidneys to damage [37]. MMP9 is integral to extracellular matrix remodelling. Previous studies have demonstrated that MMP9, as a potential renal target of DEHP with high affinity, is significant in renal cell carcinoma [38]. Molecular docking results indicated that DEHP could form stable binding with these candidate mechanistic targets. Notably, in the in vitro model of DKD induced by HG/PA, DEHP further exacerbated the abnormal expression of these candidate mechanistic targets. The trend of the MEHP effect on cells was basically consistent with that of DEHP. This suggests that DEHP and metabolic disorder factors have a synergistic effect in inducing kidney injury. In the DKD state, cells are in a state of high glucose, lipotoxicity, oxidative stress, and inflammatory activation. Under this circumstance, environmental pollutants may further disrupt the cellular homoeostasis and amplify the damage caused by metabolic stress [39]. These results predict potential targets and research directions for elucidating the molecular mechanisms underlying DEHP‐induced DKD.

### 4.1 Limitations

Although this study established a multi‐level evidence chain of ‘population association–network toxicology prediction–cell validation’, several limitations still exist. Firstly, the NHANES database is derived from a cross‐sectional observational study, which cannot definitively establish a causal relationship between DEHP exposure and DKD. Meanwhile, the exposure assessment also has certain limitations as it is unable to distinguish the specific exposure patterns of DEHP, including the source, route, frequency and duration of exposure. Secondly, although this study took sample weights into account in the statistics and corrected for various confounding factors such as age, gender, and BMI, it still cannot completely rule out the influence of unmeasured confounding factors. Furthermore, we used the multiple imputation method to handle the missing data. In cases where urine samples, renal function indicators, or covariates were missing, the final population included in the analysis might differ from the overall population. This could introduce a certain selection bias for the research subjects. Thirdly, network toxicology and molecular docking represent predictive approaches relying on databases and computational algorithms. Their outcomes are susceptible to database completeness, target annotation quality, and algorithm selection. Although we have conducted preliminary in vitro validation, mechanistic verification remains incomplete as neither protein‐level validation nor cellular functional experiments were conducted in this study. In addition, the concentrations employed in the in vitro experiments exceeded typical exposure levels in the actual environment. Accordingly, caution should be exercised when extrapolating the present findings to the general population. Future studies with lower doses or combined exposure scenarios are still needed. Based on this, animal models, multi‐cellular systems, and functional intervention experiments should be integrated. Such comprehensive approaches will help further verify the key mechanisms of DEHP and its metabolites in DKD.

## 5 Conclusion

This study showed that DEHP exposure was significantly associated with an increased risk of developing DKD. ALB, ESR1 and MMP9 were predicted to be candidate mechanistic targets of DEHP. Collectively, these findings offer preliminary evidence supporting a role for DEHP exposure in the development of DKD. However, additional prospective investigations and in‐depth mechanistic studies are still warranted.

## Author Contributions

**Dapeng Yin:** writing – original draft, investigation, conceptualization, data curation. **Yan Li:** formal analysis. **Wei Wu:** methodology. **Xiaojuan Wang:** validation. **Junhua He:** software. **Yikun Zhu:** visualization. **Jin Li:** writing – review and editing, supervision, project administration, funding acquisition.

## Funding

This work was supported by the National Natural Science Foundation of China (Grant 82270915), Shanxi Province Higher Education ‘Billion Project’ Science and Technology Guidance Project (Grant BYJL010), Special Fund for Science and Technology Innovation Teams of Shanxi Province (Grant 202304051001021), and Research Project of the Shanxi Provincial Health Commission (Grant 2024078).

## Ethics Statement

This project was approved by the Research Ethics Review Committee of the National Centre for Health Statistics and the Centres for Disease Control and Prevention. This study utilised publicly available databases for analysis and did not require additional ethical applications.

## Conflicts of Interest

The authors declare no conflicts of interest.

## Supporting information


Supporting Information S1

## Data Availability

The data that support the findings of this study are available from the corresponding author upon reasonable request.

## References

[1] K. Zhang , Y. Lin , X. Sun , et al., “Differential Life Cycle Toxic Effects and Molecular Mechanisms of Di(2‐Ethylhexyl) Phthalate (DEHP) Exposure on the Female Reproductive System,” Toxicology 518 (2025): 154275, 10.1016/j.tox.2025.154275. 40897226

[2] J. Wang , H. Liu , X. Kou , et al., “Toxic Effects of DEHP and MEHP on Gut‐Liver Axis in Rats via Intestinal Flora and Metabolomics,” iScience 27, no. 11 (2024): 111135, 10.1016/j.isci.2024.111135. 39555414 PMC11565036

[3] K. Xu , Y. Wang , X. Gao , et al., “Polystyrene Microplastics and Di‐2‐Ethylhexyl Phthalate Co‐Exposure: Implications for Female Reproductive Health,” Environmental Science and Ecotechnology 22 (2024): 100471, 10.1016/j.ese.2024.100471. 39220680 PMC11363624

[4] H. M. Koch , H. M. Bolt , R. Preuss , and J. Angerer , “New Metabolites of Di(2‐Ethylhexyl)Phthalate (DEHP) in Human Urine and Serum After Single Oral Doses of Deuterium‐Labelled DEHP,” Archives of Toxicology 79, no. 7 (2005): 367–376, 10.1007/s00204-004-0642-4. 15700144

[5] Y. Wang , H. Zhu , and K. Kannan , “A Review of Biomonitoring of Phthalate Exposures,” Toxics 7, no. 2 (2019): 21, 10.3390/toxics7020021. 30959800 PMC6630674

[6] N. Vogel , P. Schmidt , R. Lange , et al., “Current Exposure to Phthalates and DINCH in European Children and Adolescents—Results From the HBM4EU Aligned Studies 2014 to 2021,” International Journal of Hygiene and Environmental Health 249 (2023): 114101, 10.1016/j.ijheh.2022.114101. 36805185

[7] L. E. Johns , G. S. Cooper , A. Galizia , and J. D. Meeker , “Exposure Assessment Issues in Epidemiology Studies of Phthalates,” Environment International 85 (2015): 27–39, 10.1016/j.envint.2015.08.005. 26313703 PMC4648682

[8] D. M. Mérida , B. Moreno‐Franco , M. Marquès , M. León‐Latre , M. Laclaustra , and P. Guallar‐Castillón , “Phthalate Exposure and the Metabolic Syndrome: A Systematic Review and Meta‐Analysis,” Environmental Pollution 333 (2023): 121957, 10.1016/j.envpol.2023.121957. 37328121

[9] S. T. Huang , T. J. Hsieh , Y. C. Lee , et al., “Phthalate Exposure Increases Oxidative Stress, Early Renal Injury, and the Risk of Calcium Urolithiasis: A Case‐Control Study,” Ecotoxicology and Environmental Safety 287 (2024): 117322, 10.1016/j.ecoenv.2024.117322. 39547061

[10] P. C. Huang , T. Y. Lin , C. C. Wu , Y. C. Lo , W. Y. Lin , and H. B. Huang , “Relationship Between Phthalate Exposure and Kidney Function in Taiwanese Adults as Determined Through Covariate‐Adjusted Standardization and Cumulative Risk Assessment,” Ecotoxicology and Environmental Safety 285 (2024): 117091, 10.1016/j.ecoenv.2024.117091. 39341136

[11] J. Li , K. Guo , J. Qiu , et al., “Epidemiological Status, Development Trends, and Risk Factors of Disability‐Adjusted Life Years Due to Diabetic Kidney Disease: A Systematic Analysis of Global Burden of Disease Study 2021,” Chinese Medical Journal (England) 138, no. 5 (2025): 568–578, 10.1097/cm9.0000000000003428. PMC11882292 39863522

[12] F. Zuo , Y. Wang , X. Xu , et al., “CCDC92 Deficiency Ameliorates Podocyte Lipotoxicity in Diabetic Kidney Disease,” Metabolism 150 (2024): 155724, 10.1016/j.metabol.2023.155724. 37952690

[13] M. C. Thomas , M. Brownlee , K. Susztak , et al., “Diabetic Kidney Disease,” Nature Reviews Disease Primers 1 (2015): 15018, 10.1038/nrdp.2015.18. PMC7724636 27188921

[14] R. E. Dales , L. M. Kauri , and S. Cakmak , “The Associations Between Phthalate Exposure and Insulin Resistance, β‐Cell Function and Blood Glucose Control in a Population‐Based Sample,” Science of the Total Environment 612 (2018): 1287–1292, 10.1016/j.scitotenv.2017.09.009. 28898934

[15] J. W. Chang , K. W. Liao , C. Y. Huang , et al., “Phthalate Exposure Increased the Risk of Early Renal Impairment in Taiwanese Without Type 2 Diabetes Mellitus,” International Journal of Hygiene and Environmental Health 224 (2020): 113414, 10.1016/j.ijheh.2019.10.009. 31784327

[16] L. Yang , X. Liu , Z. Peng , et al., “Exposure to Di‐2‐Ethylhexyl Phthalate (DEHP) Increases the Risk of Cancer,” BMC Public Health 24, no. 1 (2024): 430, 10.1186/s12889-024-17801-w. 38341560 PMC10859012

[17] Y. He , J. Zou , T. Hong , and D. Feng , “Association Between Di‐2‐Ethylhexyl Phthalate and Nonalcoholic Fatty Liver Disease Among US Adults: Mediation Analysis of Body Mass Index and Waist Circumference in the NHANES,” Food and Chemical Toxicology 179 (2023): 113968, 10.1016/j.fct.2023.113968. 37506862

[18] American Diabetes Association , “Classification and Diagnosis of Diabetes: Standards of Medical Care in Diabetes‐2021,” supplement, Diabetes Care 44, no. S1 (2021): S15–S33, 10.2337/dc21-s002. 33298413

[19] J. Tan , J. Du , J. Liu , W. Zhao , and Y. Liu , “Prognostic Effect of Neutrophil Percentage‐to‐Albumin Ratio (NPAR) on All‐Cause and Cardiovascular Mortality in Diabetic Kidney Disease (DKD): NHANES 1999–2018,” Diabetology & Metabolic Syndrome 17, no. 1 (2025): 105, 10.1186/s13098-025-01674-z. 40148888 PMC11951754

[20] W. Yao , J. Huo , J. Ji , K. liu , and P. Tao , “Elucidating the Role of Gut Microbiota Metabolites in Diabetes by Employing Network Pharmacology,” Molecular Medicine 30, no. 1 (2024): 263, 10.1186/s10020-024-01033-0. 39707185 PMC11660459

[21] X. Yang and W. Li , “Comprehensive Analysis of the Potential Mechanism of Gansui in Blocking Non‐Small Cell Lung Cancer Progression,” Pharmaceutical Biology 63, no. 1 (2025): 170–187, 10.1080/13880209.2025.2471844. 40029169 PMC11878171

[22] C. H. Chin , S. H. Chen , H. H. Wu , C. W. Ho , M. T. Ko , and C. Y. Lin , “cytoHubba: Identifying Hub Objects and Sub‐Networks From Complex Interactome,” supplement, BMC Systems Biology 8, no. S4 (2014): S11, 10.1186/1752-0509-8-s4-s11. 25521941 PMC4290687

[23] Y. Liu , X. Yang , J. Gan , S. Chen , Z. X. Xiao , and Y. Cao , “CB‐Dock2: Improved protein‐ligand Blind Docking by Integrating Cavity Detection, Docking and Homologous Template Fitting,” Nucleic Acids Research 50, no. W1 (2022): W159–W164, 10.1093/nar/gkac394. 35609983 PMC9252749

[24] Z. Song , J. Huang , H. Zhu , et al., “Network Pharmacology, Molecular Docking, and Molecular Dynamics Simulations to Elucidate the Potential Mechanism of Ermiao San in Osteoarthritis,” Food Science and Nutrition 13, no. 12 (2025): e71287, 10.1002/fsn3.71287. 41383581 PMC12690495

[25] Y. Guo , T. Xue , X. Meng , et al., “Qihuang Yishen Formula Attenuates Renal Tubular Injury by Modulating Inflammation via the cGAS/STING Pathway in Diabetic Kidney Disease,” Phytomedicine 148 (2025): 157399, 10.1016/j.phymed.2025.157399. 41124706

[26] Q. Yu , Y. Chen , Y. Zhao , et al., “Nephropathy Is Aggravated by Fatty Acids in Diabetic Kidney Disease Through Tubular Epithelial Cell Necroptosis and Is Alleviated by an RIPK‐1 Inhibitor,” Kidney Diseases (Basel) 9, no. 5 (2023): 408–423, 10.1159/000529995. PMC10624943 37927402

[27] X. Sun , W. Zhang , Y. Wang , et al., “Combined Exposure to Di(2‐Ethylhexyl) Phthalate and Polystyrene Microplastics Induced Renal Autophagy Through the ROS/AMPK/ULK1 Pathway,” Food and Chemical Toxicology 171 (2023): 113521, 10.1016/j.fct.2022.113521. 36423728

[28] I. Lee , J. Y. Park , S. Kim , et al., “Association of Exposure to Phthalates and Environmental Phenolics With Markers of Kidney Function: Korean National Environmental Health Survey (KoNEHS) 2015–2017,” Environment International 143 (2020): 105877, 10.1016/j.envint.2020.105877. 32645486

[29] L. Trasande , S. Sathyanarayana , and H. Trachtman , “Dietary Phthalates and Low‐Grade Albuminuria in US Children and Adolescents,” Clinical Journal of the American Society of Nephrology 9, no. 1 (2014): 100–109, 10.2215/cjn.04570413. 24178978 PMC3878700

[30] H. M. Koch , H. M. Bolt , and J. Angerer , “Di(2‐Ethylhexyl)Phthalate (DEHP) Metabolites in Human Urine and Serum After a Single Oral Dose of Deuterium‐Labelled DEHP,” Archives of Toxicology 78, no. 3 (2004): 123–130, 10.1007/s00204-003-0522-3. 14576974

[31] H. M. Koch , R. Preuss , and J. Angerer , “Di(2‐Ethylhexyl)Phthalate (DEHP): Human Metabolism and Internal Exposure–An Update and Latest Results,” International Journal of Andrology 29, no. 1 (2006): 155–165, discussion 181–185, 10.1111/j.1365-2605.2005.00607.x. 16466535

[32] Q. Liu , S. Guo , Y. Tong , et al., “Renal Lipotoxicity in Diabetic and Obesity‐Associated Kidney Diseases: Molecular Mechanisms and Therapeutic Targeting,” Diabetes, Obesity and Metabolism 28, no. 1 (2026): 60–82, 10.1111/dom.70220. 41126548

[33] A. Yasuda , W. Murase , A. Kubota , et al., “Effects of Di‐(2‐Ethylhexyl) Phthalate and Its Metabolites on Transcriptional Activity via Human Nuclear Receptors and Gene Expression in HepaRG Cells,” Toxicology in Vitro 101 (2024): 105943, 10.1016/j.tiv.2024.105943. 39341470

[34] C. T. Wu , C. C. Wang , L. C. Huang , S. H. Liu , and C. K. Chiang , “Plasticizer Di‐(2‐Ethylhexyl)Phthalate Induces Epithelial‐to‐Mesenchymal Transition and Renal Fibrosis In Vitro and In Vivo,” Toxicological Sciences 164, no. 1 (2018): 363–374, 10.1093/toxsci/kfy094. 29669060

[35] M. Garoufis , S. F. Sakkou , C. E. Kostara , E. Bairaktari , and V. Tsimihodimos , “Metabolomics for Preclinical Detection of Diabetic Kidney Disease: A Comprehensive Review,” International Journal of Molecular Sciences 27, no. 2 (2026): 998, 10.3390/ijms27020998. 41596641 PMC12842068

[36] H. Shi , X. Zhao , Q. Peng , et al., “Green Tea Polyphenols Alleviate Kidney Injury Induced by Di(2‐Ethylhexyl) Phthalate in Mice,” American Journal of Nephrology 55, no. 1 (2024): 86–105, 10.1159/000534106. 37734331

[37] W. Tonnus , F. Maremonti , S. Gavali , et al., “Multiple Oestradiol Functions Inhibit Ferroptosis and Acute Kidney Injury,” Nature 645, no. 8082 (2025): 1011–1019, 10.1038/s41586-025-09389-x. 40804518 PMC12460175

[38] B. Ran , X. Wang , B. Liu , et al., “Toxicity and Mechanistic Analysis of Di(2‐Ethylhexyl)Phthalate in Renal Cell Carcinoma Progression: A Systematic Study With Network Toxicology and Molecular Docking Strategies,” Discover Oncology 16, no. 1 (2025): 1853, 10.1007/s12672-025-03543-7. 41071403 PMC12514089

[39] W. J. Ding , S. L. Huang , S. Huang , W. P. Xu , and W. Wei , “Di(2‐Ethylhexyl) Phthalate Mediates Oxidative Stress and Activates p38MAPK/NF‐kB to Exacerbate Diabetes‐Induced Kidney Injury In Vitro and In Vivo Models,” Toxicology Research (Cambridge) 12, no. 2 (2023): 332–343, 10.1093/toxres/tfad022. PMC10141783 37125328

