# Supplementary material for: Di(2‐Ethylhexyl) Phthalate Exposure and Risk of Diabetic Kidney Disease: Epidemiological Association and Mechanistic Insights
Source: Diabetes Metab Res Rev. 2026 Jul 28;42(6):e70209. doi: 10.1002/dmrr.70209 (PMC13411198; doi:10.1002/dmrr.70209)
Supplement: Supplementary file 1 — Supporting Information S1 [file DMRR-42-e70209-s001.docx]

Supplementary Table S1. The primer sequences included in the study

| Gene | Forward primer (5′-3′) | Reverse primer (5′-3′) |
| --- | --- | --- |
| **β-actin** | CACCATTGGCAATGAGCGGTTC | AGGTCTTTGCGGATGTCCACGT |
| ESR1 | GCTTACTGACCAACCTGGCAGA | GGATCTCTAGCCAGGCACATTC |
| ALB | TTTATGCCCCGGAACTCCTTT | AGTCTCTGTTTGGCAGACGAA |
| MMP9 | GCCACTACTGTGCCTTTGAGTC | CCCTCAGAGAATCGCCAGTACT |

Supplementary Table S2. Baseline characteristics of study participants according to DKD status

| Variables | Total  (n = 2149） | Non-DKD  (n = 1320） | DKD  (n = 829） | *P* |
| --- | --- | --- | --- | --- |
| Age (years) | 59.40 ± 0.41 | 56.94 ± 0.47 | 64.16 ± 0.59 | <0.001 |
| BMI (kg/m^2^) | 32.90 ± 0.24 | 32.85 ± 0.30 | 33.00 ± 0.39 | 0.760 |
| *Gender (%)* |  |  |  | 0.300 |
| Female | 1018 (47.63) | 637 (46.49) | 381 (49.83) |  |
| Male | 1131 (52.37) | 683 (53.51) | 448 (50.17) |  |
| *Race (%)* |  |  |  | 0.620 |
| Mexican American | 390 (9.27) | 250 (9.09) | 140 (9.62) |  |
| Non-Hispanic Black | 535 (14.19) | 324 (13.49) | 211 (15.55) |  |
| Non-Hispanic White | 767 (63.01) | 442 (63.99) | 325 (61.12) |  |
| Other Hispanic | 234 (5.64) | 153 (5.70) | 81 (5.54) |  |
| Other Races | 223 (7.88) | 151 (7.73) | 72 (8.17) |  |
| *Marital status (%)* |  |  |  | <0.001 |
| Married/Living with Partner | 1283 (63.80) | 841 (68.20) | 442 (55.29) |  |
| Never married | 204 (8.71) | 134 (9.38) | 70 (7.42) |  |
| Widowed/Divorced/Separated | 662 (27.49) | 345 (22.42) | 317 (37.29) |  |
| *Education level (%)* |  |  |  | 0.030 |
| Above high school | 899 (50.91) | 578 (53.89) | 321 (45.15) |  |
| Below high school | 755 (23.65) | 433 (21.87) | 322 (27.10) |  |
| High school graduate | 495 (25.44) | 309 (24.24) | 186 (27.76) |  |
| *PIR (%)* |  |  |  | 0.003 |
| <1.3 | 760 (24.27) | 447 (22.12) | 313 (28.43) |  |
| 1.3-3.5 | 832 (37.68) | 501 (36.68) | 331 (39.60) |  |
| ≥3.5 | 557 (38.06) | 372 (41.20) | 185 (31.97) |  |
| *Smoking status (%)* |  |  |  | 0.830 |
| Former | 720 (34.16) | 421 (33.88) | 299 (34.70) |  |
| Never | 1072 (49.65) | 672 (49.52) | 400 (49.92) |  |
| Current | 357 (16.19) | 227 (16.60) | 130 (15.39) |  |
| *Alcohol use (%)* |  |  |  | 0.020 |
| Former | 630 (25.13) | 359 (23.20) | 271 (28.87) |  |
| Heavy | 258 (11.94) | 166 (12.78) | 92 (10.33) |  |
| Mild | 655 (37.24) | 424 (38.17) | 231 (35.44) |  |
| Moderate | 223 (11.81) | 146 (13.25) | 77 (9.03) |  |
| Never | 383 (13.87) | 225 (12.60) | 158 (16.33) |  |
| *Hypertension (%)* |  |  |  | <0.001 |
| No | 614 (29.00) | 456 (35.18) | 158 (17.04) |  |
| Yes | 1535 (71.00) | 864 (64.82) | 671 (82.96) |  |
| *Hyperlipidemia (%)* |  |  |  | 0.55 |
| No | 273 (11.30) | 183 (11.66) | 90 (10.61) |  |
| Yes | 1876 (88.70) | 1137 (88.34) | 739 (89.39) |  |
| *MetS (%)* |  |  |  | <0.001 |
| No | 736 (31.90) | 503 (35.20) | 233 (25.50) |  |
| Yes | 1413 (68.10) | 817 (64.80) | 596 (74.50) |  |
| *CVD (%)* |  |  |  | <0.001 |
| No | 1601 (75.47) | 1070 (81.19) | 531 (64.39) |  |
| Yes | 548 (24.53) | 250 (18.81) | 298 (35.61) |  |
| WBC (10^9^/L) | 7.83 ± 0.09 | 7.72 ± 0.10 | 8.02 ± 0.15 | 0.080 |
| Hb (g/dL) | 14.07 ± 0.05 | 14.21 ± 0.07 | 13.79 ± 0.08 | <0.001 |
| PLT (10^9^/L) | 242.04 ± 2.22 | 245.69 ± 2.41 | 234.98 ± 3.36 | 0.003 |
| ALT (U/L) | 27.89 ± 0.58 | 28.73 ± 0.71 | 26.27 ± 0.83 | 0.010 |
| AST (U/L) | 26.94 ± 0.45 | 26.93 ± 0.49 | 26.96 ± 0.73 | 0.980 |
| TC (mmol/L) | 4.83 ± 0.04 | 4.89 ± 0.05 | 4.72 ± 0.06 | 0.040 |
| TG (mmol/L) | 2.20 ± 0.07 | 2.11 ± 0.08 | 2.37 ± 0.10 | 0.040 |
| HbA1c (%) | 7.08 ± 0.05 | 6.88 ± 0.05 | 7.45 ± 0.09 | <0.001 |
| MEHP (ng/ml) | 3.07 ± 0.28 | 3.37 ± 0.39 | 2.48 ± 0.37 | 0.100 |
| MEHHP (ng/ml) | 23.83 ± 1.92 | 24.84 ± 2.58 | 21.89 ± 2.24 | 0.370 |
| MEOHP (ng/ml) | 14.12 ± 1.03 | 14.47 ± 1.36 | 13.43 ± 1.32 | 0.570 |
| MECPP (ng/ml) | 34.87 ± 2.57 | 35.92 ± 3.45 | 32.82 ± 2.98 | 0.480 |
| Ucr (mg/dL) | 116.40 ± 2.16 | 120.30 ± 2.81 | 108.86 ± 2.68 | 0.002 |
| ln∑DEHP ng/mg crt | 3.81 ± 0.03 | 3.77 ± 0.04 | 3.88 ± 0.04 | 0.070 |


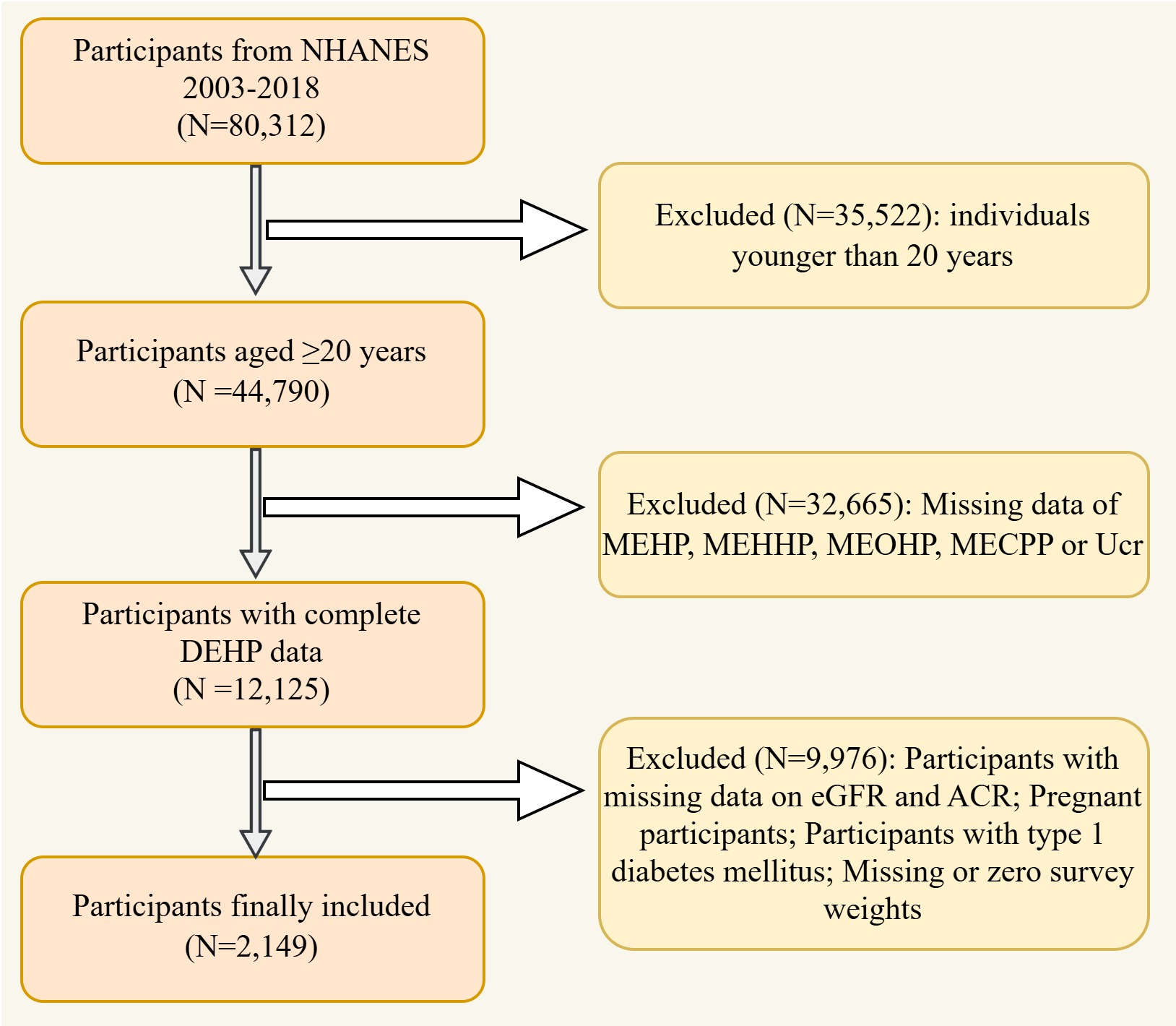


**Supplementary Figure S1.** Flowchart of screening process for NHANES participants from 2003 to 2018.


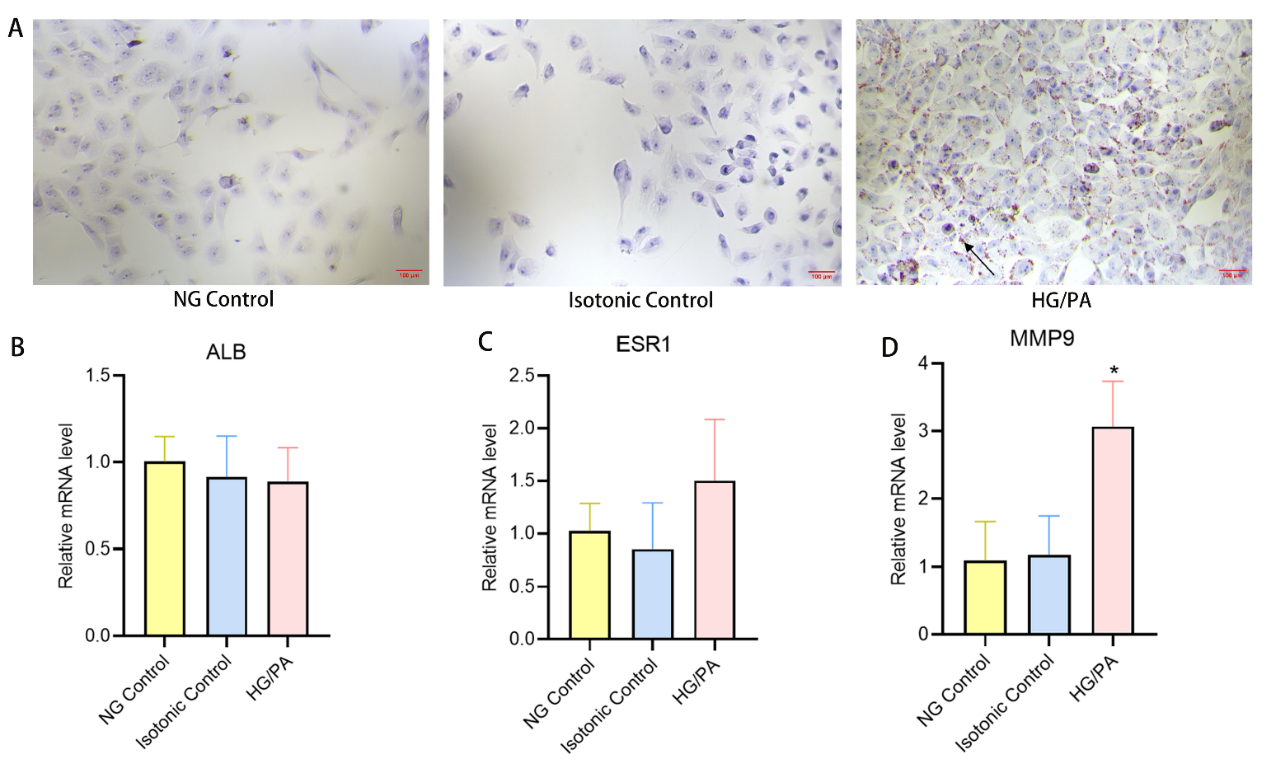


**Supplementary Figure S2. Validation of the isotonic control in HK-2 cells.**

(A) Oil Red O staining of HK-2 cells in the NG control, isotonic control, and HG/PA groups.

(B–D) Relative mRNA expression levels of (B) ALB, (C) ESR1, and (D) MMP9 in HK-2 cells cultured under NG control, isotonic control, and HG/PA conditions.

Data are presented as the mean ± SEM (n = 4). **P* < 0.05, ***P* < 0.01 versus the NG Control group.

**Abbreviations:** ALB, albumin; ESR1, estrogen receptor 1; MMP9, matrix metallopeptidase 9; HG/PA, 30 mM high glucose + 300 µM palmitic acid; NG Control, 5.6 mM glucose.
